# [Corrigendum] RNA interference-mediated depletion of TRPM8 enhances the efficacy of epirubicin chemotherapy in prostate cancer LNCaP and PC3 cells

**DOI:** 10.3892/ol.2025.14975

**Published:** 2025-03-12

**Authors:** Tao Liu, Yixiang Liao, Huangheng Tao, Jinmin Zeng, Gang Wang, Zhonghua Yang, Yongzhi Wang, Yu Xiao, Jiajie Zhou, Xinghuan Wang

Oncol Lett 15: 4129–4136, 2018; DOI: 10.3892/ol.2018.7847

Subsequently to the publication of this paper [and a corrigendum that was published to take account of errors that were made with the assembly of flow cytometric plots shown in Fig. 3A (see doi.org/10.3892/ol.2022.13211)], an interested reader drew to the attention of the Editor that the control β-actin RT-qPCR data in Fig. 1A and certain of the western blot data shown in Fig. 4C were strikingly similar to data that had already appeared in different form in articles that were published in the journals *Oncology Letters* and *Oncotarget* respectively, albeit these papers featured various of the abovementioned authors.

Upon examining their original raw data (which were also presented to the Editorial Office for our inspection), the authors realized how these errors occurred, and the revised versions of Figs. 1 and 4, containing the correct control β-actin RT-qPCR data in Fig. 1A and western blot data in Fig. 4C, are shown on the next page. The authors regret the errors that were made during the compilation of the original figures, and are grateful to the editor of *Oncology Letters* for allowing them the opportunity to publish a further Corrigendum. Furthermore, they apologize to the readership for any inconvenience caused.

## Figures and Tables

**Figure 1.**
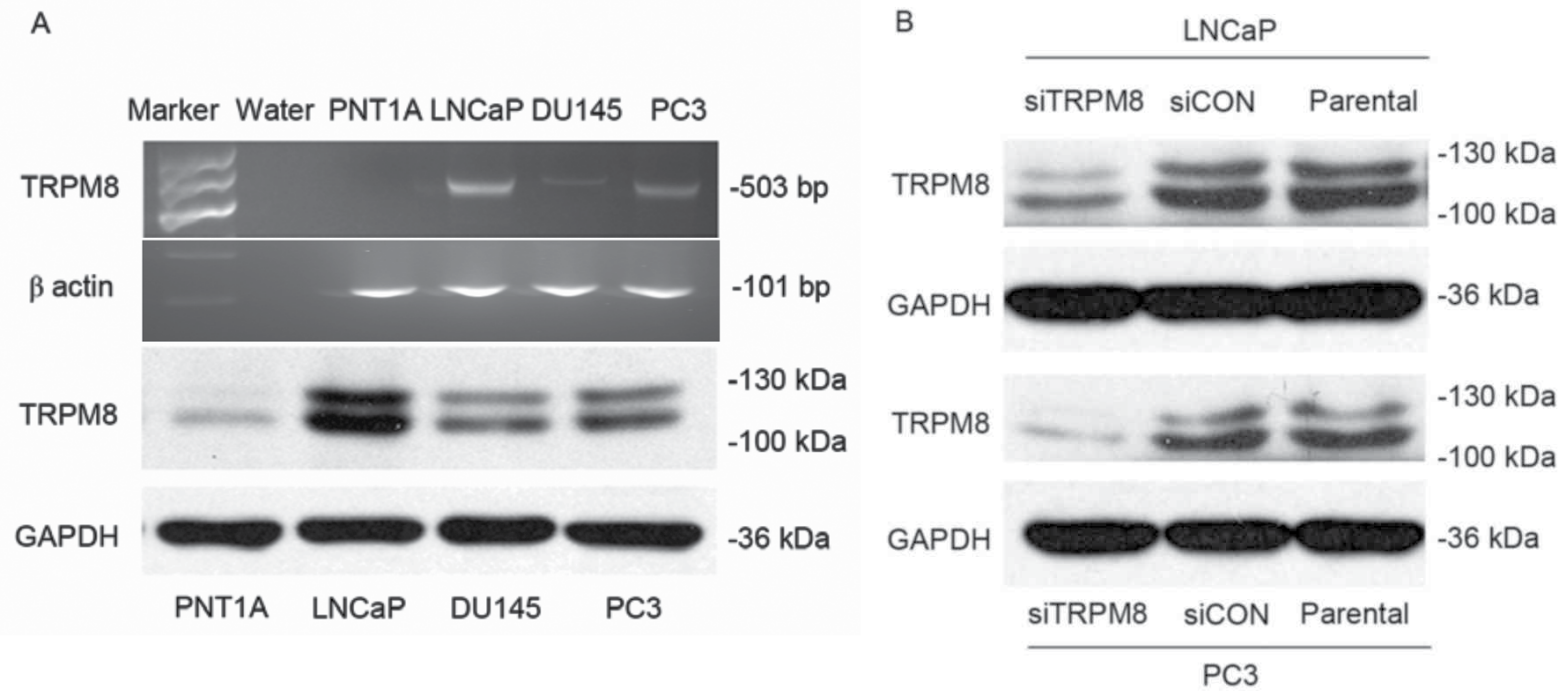
Expression of TRPM8 in cancerous and non-cancerous prostate cells and in siTRPM8 and siCON cells. (A) The expression of TRPM8 in PNT1A, LNCaP, DU145, and PC3 cells was detected by RT-PCR (above) and western blot (lower). β-actin and GAPDH were used as an internal standard in RT-PCR and western blot analysis, respectively. (B) The knockdown efficiency of siTRPM8 in LNCaP and PC3 cells. TRPM8 expression was evidently decreased in siTRPM8 cells compared with the parental and siCON cells. siTRPM8, small interfering RNA targeting transient receptor potential cation channel subfamily M member 8; CON, negative control.

**Figure 4.**
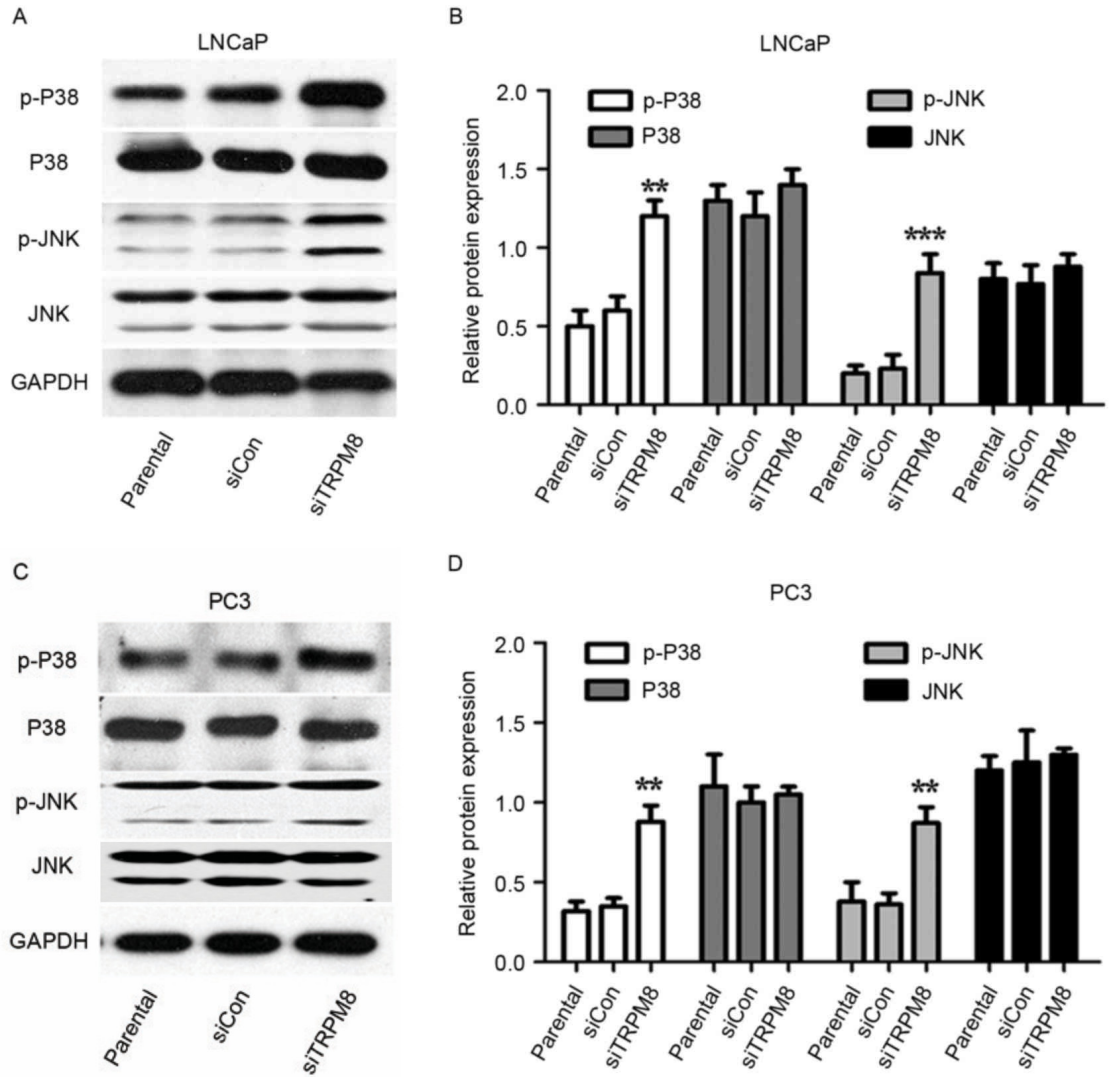
Mitogen-activated protein kinase signal pathways may partially involve in the sensitization activity of siTRPM8 towards LNCaP and PC3 cells. (A-D) Western blot analysis in (A) LNCaP cells, with (B) quantification and in (C) PC3 cells, with (D) quantification. This analysis was performed performed to investigate the expression of p-p38, p38, p-JNK, and JNK in parental, siCON, siTRPM8 cells. **P<0.01, ***P<0.001, compared with the parental group. p-p38, phosphorylated p38 mitogen-activated protein kinase; JNK, c-Jun N-terminal kinase; siTRPM8, small interfering RNA targeting transient receptor potential cation channel subfamily M member 8; CON, negative control.

